# Fluorinated PEG-PEI Coated Magnetic Nanoparticles for siRNA Delivery and CXCR4 Knockdown

**DOI:** 10.3390/nano12101692

**Published:** 2022-05-16

**Authors:** Yixiang Cao, Shiyin Zhang, Ming Ma, Yu Zhang

**Affiliations:** 1 State Key Laboratory of Bioelectronics, Jiangsu Key Laboratory for Biomaterials and Devices, School of Biological Sciences and Medical Engineering, Southeast University, Nanjing 210096, China; 220195143@seu.edu.cn; 2 Nanjing Nanoeast Biotech Co., Ltd., Nanjing 211000, China; syzhang@nanoeast.net

**Keywords:** magnetic nanoparticles, fluorination, polyethyleneimine, polyethylene glycol, RNA interference, CXC chemokine receptor 4

## Abstract

CXC chemokine receptor 4 (CXCR4) is a promising therapeutic target. Previous studies have shown that intracellular delivery of siRNA to knockdown CXCR4 expression in cancer cells is an effective therapeutic strategy. To prepare efficient magnetic nucleic acid carriers, it is now necessary to improve the endocytosis efficiency of PEGylated magnetic nanoparticles. In our work, Heptafluorobutyryl-polyethylene glycol-polyethyleneimine (FPP) was first prepared and then used to coat magnetic nanoparticles (MNPs) to obtain magnetic nanocarriers FPP@MNPs. The materials were characterized by ^19^ F-Nuclear Magnetic Resonance (NMR), transmission electron microscope (TEM), energy dispersive spectroscopy (EDS), and dynamic light scattering (DLS). The biosecurity of FPP@MNPs was confirmed by cell viability and apoptosis experiments. Cellular uptake of FPP@MNPs and siRNA transfection enhanced by external magnetic fields were detected by fluorescence microscopy, confocal laser microscopy, and flow cytometry. The results show that the cellular uptake efficiency of FPP@MNPs was significantly improved, and transfection efficiency reached more than 90%. The knockdown of CXCR4 on the 4 T1 cell membrane was confirmed by real-time polymerase chain reaction (RT-PCR) and flow cytometry. In conclusion, the fluorinated cationic polymer-coated magnetic nanoparticles FPP@MNPs can be loaded with siRNA to reduce CXCR4 expression as well as be expected to be efficient universal siRNA carriers.

## 1. Introduction

The CXC chemokine receptor 4 (CXCR4) has become a very promising target for tumor therapies. The expression of chemokine receptors varies among cancer cells of different origins, but CXCR4 is widely expressed in human cancers [1,2,3,4,5].

The interaction between CXC chemokine ligand 12/chemokine stromal cell-derived factor-1 (CXCL12/SDF-1) and the corresponding receptor CXCR4 promotes tumor metastasis [6]. Furthermore, CXCR4 overexpression is associated with poor prognosis in many cancer subtypes. As is reported, CXCR4 is highly expressed in breast cancers, with the ligand CXCL12 showing the highest levels of expression in the organ where cancer metastases first occur [7]. Moreover, CXCL12 activating CXCR4 promotes the transfer of proto-cancer mitochondria between cells [8]. Activation of CXCR4 promotes trafficking and homing of acute myeloid leukemia cells to the bone marrow and spleen [9]. The CXCL12-CXCR4 axis is one of the most important factors in tumor metastasis, angiogenesis, and drug resistance. Therefore, it is of great significance to develop efficient methods to block CXCR4 in tumors.

Currently, studies of medicine against CXCR4 focus on inhibitors or antagonists, such as Plerixafor (AMD3100) [10], T140 peptide [11], and E5 peptide [12]. RNA interference (RNAi) refers to the highly conserved phenomenon of efficient and specific degradation of homologous mRNA induced by double-stranded RNA (dsRNA). The applications of RNAi are generally mediated by small interfering RNA (siRNA) or micro-RNA (miRNA) [13]. The main difference between siRNA and miRNA is that siRNA specifically degrades the target mRNA, while miRNA can regulate the degradation of various mRNA sequences [14,15,16]. Since RNAi can specifically reduce the expression of target genes, it has been widely used to explore the fields of gene function and disease treatment. RNAi mediated by siRNA would be an effective strategy for reducing or even silencing CXCR4 expression [17].

In general, the delivery of siRNA requires carriers, so the development of efficient siRNA carriers is an important part of RNAi technology. In many studies, cationic polymer-modified magnetic nanoparticles were used for nucleic acid delivery. As is reported, polymers such as Polyethyleneimine (PEI) [18], Poly-L-lysine (PLL) [19], and chitosan [20] coated magnetic nanoparticles were used as magnetic carriers. In particular, external magnetic fields have been widely demonstrated to increase the transfection capacity of magnetic nanoparticles loaded with drugs [21]. Therefore, in our work, we focus on the development of magnetic nanocarriers that can efficiently deliver siRNA into cells based on PEI-modified magnetic nanoparticles (PEI@MNP) and verify the transfection effect.

Polyethylene glycol (PEG) has the advantages of good aqueous solubility and high biocompatibility, which makes it suitable for biomedical applications [22]. Currently, PEG is widely used to prepare biomedical materials such as liposomes [23,24], nanoparticles [25,26,27], and hydrogels [28,29]. To improve the stability and safety of PEI@MNP in a physiological environment, polyethylene glycol (PEG) modification is necessary. As is reported, PEGylation of cationic polymeric coated nucleic acid carriers can improve stability and reduce toxicity [30]. Compared with PEI@MNP, PEG-PEI@MNP has a lower positive surface charge, significantly improved biocompatibility, and reduced levels of oxidative stress and cell membrane damage [31]. PEGylation of nucleic acid carriers can effectively prevent protein adsorption and can help these carriers escape the reticuloendothelial system (RES) while maintaining prolonged blood circulation [32].

However, PEGylation of the nanoparticles would inhibit cellular uptake, and the subsequent endosomal escape efficiency would be significantly reduced [33,34]. This phenomenon is known as the “PEG dilemma”. Several approaches have been reported to overcome the "PEG dilemma". Using cleavable chemical bonds, such as hydrazide-hydrazone bonds [35], Schiff base bonds [36], thioketal bonds [37], and matrix metalloproteinase substrates [38] to connect PEG and carriers would be effective strategies to overcome this dilemma. In addition, the nanoparticles can also be modified with peptides (e.g., RGD peptide [39,40], TAT peptide [41]) to improve the efficiency of cellular uptake.

Fluoroalkyl chains are both hydrophobic and lipophobic [42]. Many studies show that fluorination is an effective strategy to improve the transfection efficiency of nucleic acid delivery vectors. As is reported, fluorinated nucleic acid carriers combine the features of liposomes and cationic polymers [43]. Fluorination enhances cellular uptake of dendrimer/DNA complexes and facilitates endosomal escape [44]. Furthermore, compared with bioreducible poly(amido amine)s (bPAA) fluorinated with trifluoroacetic anhydride or pentafluoropropionic anhydride, heptafluorobutyric anhydride-modified bPAA achieved better gene silencing effect [45]. As fluorination to overcome the “PEG dilemma” has not been reported in previous studies, we propose that fluorination may be an effective strategy to overcome this dilemma and improve the cellular uptake efficiency of PEGylated nanomaterials.

Herein, we developed a fluorinated PEG-PEI modified MNP for in vitro siRNA delivery. This fluorinated magnetic nanocarrier has low cytotoxicity and can effectively overcome the “PEG dilemma”. At the same time, high transfection efficiency can be obtained under the action of external magnetic fields, and the siRNA delivery efficiency was validated in a variety of cell lines. Furthermore, fluorinated PEG-PEI@MNP were used to deliver CXCR4 siRNA(siCXCR4) into 4 T1 cells, which could effectively reduce the expression of CXCR4 on the cell membrane.

## 2. Materials and Methods

### 2.1. Materials

Ferric acetylacetonate (AR, Sinopharm Chemical Reagent Co. Ltd., SCRC, Shanghai, China), oleic acid (85%, Aladdin, Shanghai, China), benzyl ether (98%, Sigma-Aldrich, St. Louis, MS, USA), ethanol (AR, SCRC), n-hexane (97%, SCRC), dimercaptosuccinic acid (98%, TCI, Shanghai, China), acetone (AR, SCRC), triethylamine (AR, Aladdin, Shanghai, China), methanol (AR, SCRC), polyethyleneimine (Mw 25000, Sigma-Aldrich, St. Louis, MS, USA), heptafluorobutyric anhydride (97%, Macklin, Shanghai, China), amino-polyethylene glycol-carboxyl (Mw 2000, Xi’an Ruixi, Xi’an, China), methoxy-polyethylene glycol-polyethyleneimine (mPEG-PEI, Mw 2000-25000, Xi’an Ruixi Co. Ltd., Xi’an, China) FITC (Beyotime, Shanghai, China), CCK8 kit (KeyGen, Nanjing, China), Hoechst33342 (KeyGen, Nanjing, China), rhodamine-phalloidin (KeyGen, Nanjing, China), LysoTracker (KeyGen, Nanjing, China), Annexin V-FITC/PI kit (KeyGen, Nanjing, China), Anti-CXCR4 antibody (Abcam of Thermo Fisher Scientific Inc., Waltham, MA, USA), DMEM high glucose medium (KeyGen, Nanjing, China), fetal bovine serum (Gibco of Thermo Fisher Scientific Inc., Waltham, MA, USA), trypsin (Gibco of Thermo Fisher Scientific Inc., Waltham, MA, USA), siRNA(GenePharma Co. Ltd., Shanghai, China), 4 T1, A549, HeLa cell line was provided by Southeast University School of Medicine (Nanjing, China).

### 2.2. Methods

#### 2.2.1. Synthesis of Fluorinated Polyethylene Glycol-Polyethyleneimine (F_7_-PEG-PEI)

**Figure 1:**
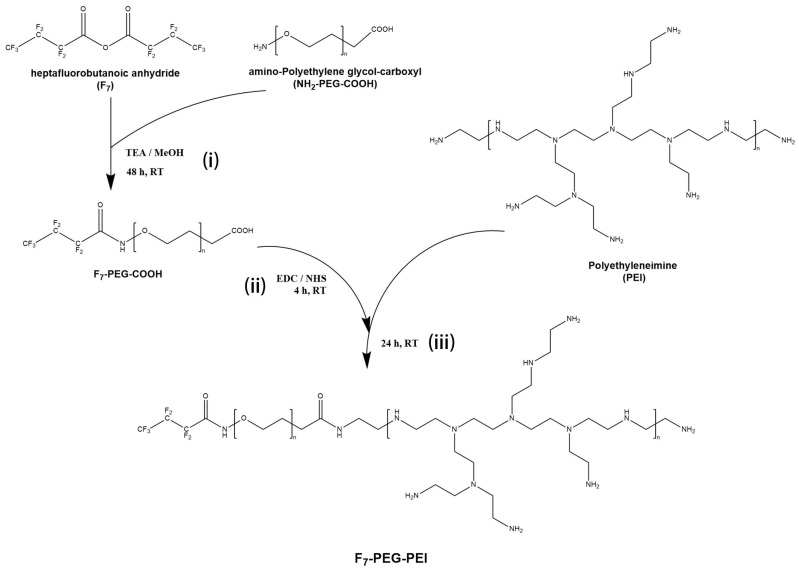
Synthetic process of F_7_-PEG-PEI. TEA means triethylamine, MeOH means methanol, RT means room temperature.

The synthesis steps of F_7_-PEG-PEI (FPP) are shown in Figure 1.

Step (i):

(1) 200 μmol of heptafluorobutyric anhydride (F_7_) and 100 μmol of amino-polyethylene glycol-carboxyl (NH_2_-PEG-COOH) were dissolved in 10 mL methanol, catalyzed by 50 μL triethylamine, magnetic stirred at 240 rpm, and reacted at room temperature for 48 h.;
(2) Then, dialysis membrane (1200 MWCO) was used, and the product was dialyzed against ultrapure water for 3 days. After dialysis, a rotary evaporator was used to evaporate most of the water. Vacuum drying was performed for 48 h to obtain F_7_-PEG-COOH.

Step (ii):

(3) 100 mg of F_7_-PEG-COOH was dissolved in 50 mL pure water. Then 20 mg of 1-(3-dimethylaminopropyl)-3-ethylcarbodiimide hydrochloride (EDC) and 10 mg of N-hydroxysuccinimide (NHS) were added, and the reaction was stirred at room temperature for 2 h.

Step (iii):

(4) Then, 1200 mg of polyethyleneimine was added, and the reaction was stirred at room temperature for 48 h;
(5) Dialysis membrane (8000 MWCO) was used, and the product was dialyzed against pure water for 3 days;
(6) After dialysis, a rotary evaporator was used to evaporate most of the water. Vacuum drying was performed for 48 h to obtain F_7_-PEG-PEI.

#### 2.2.2. Synthesis of Fe_3_O_4_ Magnetic Nanoparticles

Firstly, 0.7 g (2 mmol) iron acetylacetonate (Fe(acac)₃), 20 mL of benzyl ether, and 30 mL of oleic acid were mixed. Then, nitrogen gas was introduced below the liquid level, and the mixture was condensed and refluxed without stirring while constantly bubbling nitrogen through the reaction mixture. Solution was heated from room temperature to 220 °C at a rate of 3.3 °C/min and kept at 220 °C for 60 min. Then, the temperature was raised to 290 °C at a rate of 3.3 °C/min and kept for 30 min. After the solution was naturally cooled to room temperature, absolute ethanol was added to the system to wash the products, followed by magnetic separation for 5 min to discard the supernatant, and repeat 3 times. The oleic acid-modified Fe_3_O_4_ magnetic nanoparticles (OA@MNPs) were prepared and were dissolved in 100 mL of n-hexane. The Fe concentration of OA@MNPs determined by UV-Vis spectrophotometry (Method S2) was 8.22 mg/mL suspension, and the yield was 74%.

The suspension of OA@MNPs (containing 800 mg of Fe) in 100 mL of hexane as described above was added to a solution of dimercaptoscuccinic acid (DMSA, 400 mg) in acetone (200 mL). The mixture was condensed and refluxed for 4 h in water bath at 60 °C under electric stirring at 500 rpm. After that, 200 mL of ultrapure water was added to extract the product. The product was magnetically separated and the supernatant was discarded. Then, 200 mL of pure water was added to wash the product. The product was magnetically separated and the supernatant was discarded, repeated 3 times. The products were additionally subjected to dialysis (48 h, MWCO 10000) and kept in aqueous suspension (100 mL of water) to obtain DMSA@MNPs. The Fe concentration was 5.11 mg/mL suspension, measured by UV-Vis spectrophotometry (Shimadzu Inc., Kyoto, Japan) (Method S2), with a yield of 64% based on Fe content.

#### 2.2.3. Preparation of F_7_-PEG-PEI Coated MNPs (FPP@MNPs)

The suspension of DMSA@MNPs (containing 5 mg Fe) and 20 mg of FPP were mixed in 40 mL of pure water, and the mixture was reacted under electric stirring at 500 rpm with 100 W ultrasonic vibration for 1 h. The solution was then purified by ultrafiltration (100 kDa NMWL) 6 times and kept in aqueous suspension (3 mL of water). The Fe concentration of FPP@MNPs was 1.17 mg/mL, measured by UV-Vis spectrophotometry, with a yield of 70% based on Fe content. FPP@MNPs were characterized by magnetization, TEM, EDS, DLS, and zeta potential. mPEG-PEI coated MNPs were prepared by Method S1. Size distribution of mPEG-PEI@MNPs was shown in Figure S5.

#### 2.2.4. Cell Viability and Apoptosis

(1) Cell viability was detected by CCK8 kit. HeLa cells were cultured in a 96-well plate, 10^4^ cells per well.100 μL of culture medium (DMEM and 10% FBS) was added per well, and cells were cultured at 37 °C, 5% CO_2_ for 16 h. After that, the culture medium was replaced, and FPP@MNPs were added at the final concentrations of 2, 4, 6, 8, 10, and 12 μg/mL. Cells were incubated for 24 or 48 h. Then 10 μL of CCK-8 solution was added to each well, and the OD value was detected at λ = 450 nm after incubation for 1 h.
  The cell viability was calculated according to the following formula:
  Cell viability (%) = [(As − Ab)/(Ac − Ab)] × 100;
  As = experimental well absorbance (cells, medium, CCK-8 and FPP@MNPs);
  Ab = absorbance of blank wells (medium and CCK-8);
  Ac = control well absorbance (cells, medium and CCK-8).

(2) Cell apoptosis was detected by Annexin V-FITC/PI apoptosis kit. HeLa cells were cultured in a 6-well plate, 5 × 10^5^ cells per well. In total, 2 mL of culture medium (DMEM and 10% FBS) was added per well, and cells were cultured at 37°C, 5% CO_2_ for 16 h. Then FPP@MNPs were added at the final concentrations of 2.5 or 5 μg/mL. Cells were incubated for 24 or 48 h. After the cells were digested with 0.25% trypsin solution, Annexin V-FITC and PI were added and measured by flow cytometry Attune NxT (Invitrogen Inc., Carlsbad, CA, USA).

#### 2.2.5. Observation of Cellular Uptake and Transfection by Laser Confocal Microscopy

(3) Cells were cultured in glass-bottom dishes with a diameter of 35 mm and a thickness of 0.17 mm, with 5 × 10^5^ cells per dish, and the volume of the culture medium was 2 mL.
(4) Preparation of FITC-labeled(green) nanoparticles:
  1 mg of F_7_-PEG-PEI@MNPs (FPP@MNPs) or mPEG-PEI@MNPs (mPP@MNPs) and 50 μg FITC were mixed at room temperature and placed in the dark for 24 h. After that, the ultrafiltration purification (100 kDa NMWL) was repeated three times.
(5) Observation of cellular uptake:
  FITC-labeled FPP@MNP or mPP@MNP (containing 10 μg Fe) was mixed with 2 mL DMEM medium containing LysoTracker (red) and Hoechst33342 (blue). Then the mixture was added to the cells, placed under a confocal microscope for observation.
(6) Observation of cell transfection:
  The transfected cells were added to a 2.5% glutaraldehyde solution, fixed at 4 °C for 30 min, and then washed three times with PBS. F-actin was stained with rhodamine-phalloidin (red), and nuclei were stained with Hoechst33342. siRNA labeled with FAM (green).
(7) The excitation wavelength:
  405 nm for blue fluorescence, 488 nm for green fluorescence, and 562 nm for red fluorescence.

#### 2.2.6. Cell Transfection

Cells were cultured in 24-well plates, 1 × 10^5^ cells per well, with 500 μL DMEM medium (10% FBS), incubated at 37 °C, 5% CO_2_ until the cell density reaches 60%.

External magnetic field-enhanced transfection (MagTrans) used the following steps:

(1) The medium in each well was replaced with fresh 500 μL of DMEM medium (10% FBS);
(2) For each well, 40 pmol of siRNA was dissolved in 10 μL of DEPC water, and then mixed with 0.25, 0.5, 0.75, or 1.0 μg of FPP@MNP (calculated as Fe content). Incubated at room temperature for 10–15 min to obtain FPP@MNP/siRNA complexes;
(3) The above complexes were added to the medium in the well;
(4) The 24-well plate was placed on the magnetic plate (400 mT) and incubated at 37 °C for 10–20 min;
(5) The magnetic plate was removed, and the cells were cultured at 37 °C, 5% CO_2_.

#### 2.2.7. RT-PCR

RNA was extracted from the transfected cells using Trizol (Invitrogen). RNA concentration was detected by Nanodrop spectrophotometer (Thermo Fisher Scientific Inc., Waltham, MA, USA). Both reverse transcription and SYBR green fluorescence quantitative PCR were performed on LightCycler96 (Roche Inc., Basel, Switzerland) using TB Green Premix Ex Taq II kit (Takara Bio Inc., Kusatsu, Japan). The primer sequences are shown in the table below. The relative expression of CXCR4 was calculated with Actin as reference. The sequences of primer are shown in Table 1.

#### 2.2.8. Transfection Efficiency and CXCR4 Expression Measured by Flow Cytometry

Cells were digested with 0.25% trypsin solution, centrifuged at 1000 rpm, and washed three times with PBS. Cells were transfected with FAM-labeled RNA with excitation light of 488 nm wavelength and detection channel of 530 nm wavelength.

Cells transfected with siCXCR4 were fixed with 4% paraformaldehyde solution at 4 °C for 30 min. Then cells were centrifuged at 1000 rpm and washed for 3 times with PBS. In total, 5 × 10^5^ cells were mixed with 0.5 μg Cy5-labeled Anti-CXCR4 antibody and then incubated at room temperature for 30 min. Then the cells were centrifuged at 1000 rpm and washed for 3 times with PBS. An excitation light of 637 nm wavelength and a detection channel of 695 nm wavelength were used.

#### 2.2.9. Characterization

Elements’ content measured by energy dispersive spectroscopy (EDS): 50 μL of F_7_-PEG-COOH solution was dropped on a single-crystal silicon wafer (0.5 cm × 0.5 cm). After drying at room temperature, the silicon wafers were placed in scanning electron microscope (Ultra Plus, Zeiss Inc., Oberkochen, Germany) for characterization.

Nanoparticles observed by transmission electron microscope (TEM): 20 μL of OA@MNPs, DMSA@MNPs, or FPP@MNPs were dropped on a 200-mesh copper grid. The copper grid was dried at room temperature and then placed in a TEM for observation. HRTEM imaging and SAED were performed on JEM-2100 (JEOL Ltd., Akishima, Japan) at an operating voltage of 200 kV. EDS mapping was performed on FEI Tecnai G2 F30(FEI Inc., Hillsboro, OR, USA) at an operating voltage of 300 kV.

Magnetization curves: The OA@MNPs (containing 20 mg Fe) were dried and put into vibrating sample magnetometer (Lakeshore 7407, Lakeshore Inc., Columbus, OH, USA) for measurement. Set the magnetic field range from −7500 G to 7500 G.

^19^ F-Nuclear Magnetic Resonance (NMR): 20 mg of F_7_-PEG-PEI was dissolved in 0.5 mL of CD_3_ OD and using Bruker 600M (Bruker Inc., Karlsruhe, Germany) for characterization, the operating frequency was 600 MHz.

Characterization of size and zeta potential: the size distribution and zeta potential of DMSA@MNPs or FPP@MNPs were measured by Zetasizer ZS90 (Malvern Panalytical Ltd., Malvern, UK). The sample was diluted with pure water to a final concentration of 0.1 mg/mL Fe, and then 1 ml of the sample was added to the sample pool before testing. DLS distribution of mPEG-PEI@MNPs is shown in Figure S5.

**Figure 2:**
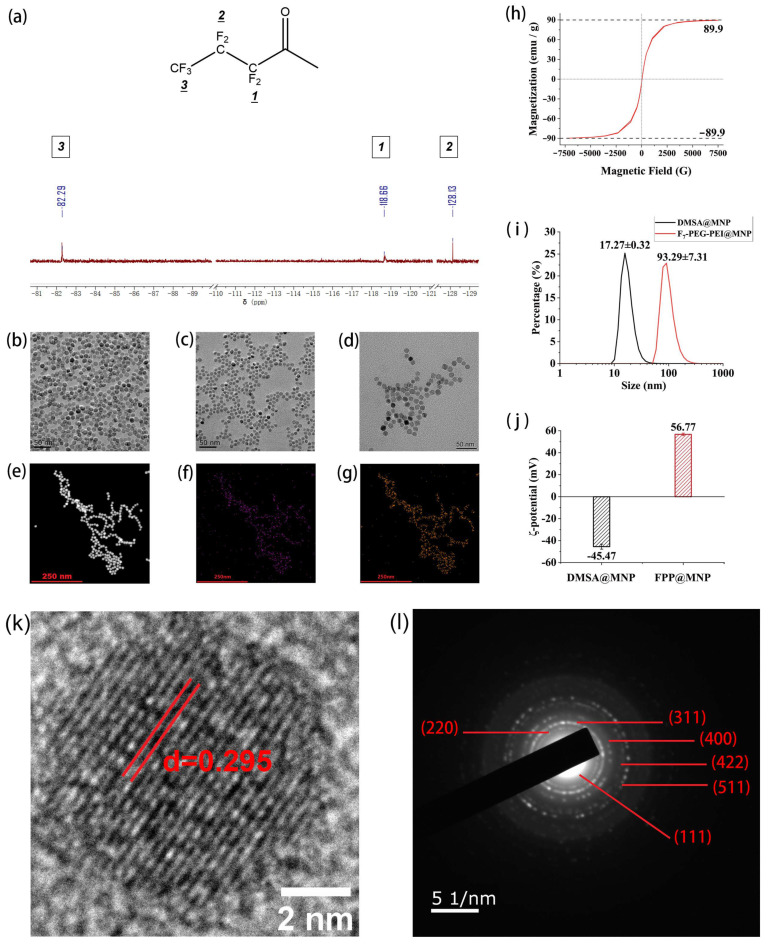
(**a**) ^19^ F-NMR spectrum of F_7_-PEG-PEI (FPP). (**b**) TEM micrograph of oleic acid-modified MNPs. (**c**) TEM micrograph of DMSA@MNP. (**d**) TEM micrograph of FPP@MNP. (**e**) HAADF micrograph of FPP@MNP. (**f**) Distribution of F element of FPP@MNP. (**g**) Distribution of Fe element of FPP@MNP. (**h**) Magnetization curve of MNPs. (**i**) Hydrodynamic size distribution (intensity) of DMSA@MNP and FPP@MNP. (**j**) Zeta potential of DMSA@MNP and FPP@MNP. (**k**) HRTEM image of oleic acid-modified MNPs. (**l**) SAED image of oleic acid-modified MNPs.

50 particles in Figure 2b were randomly selected for TEM size measurement by ImageJ. Figure S1 shows the TEM size distribution. Figure S2 shows the filtered HRTEM image for measuring lattice spacing of OA@MNPs. Figure S3 shows the absorbance standard curve for measuring Fe concentration.

### 2.3. Statistical Analysis

The data were expressed as the mean ± standard deviation (SD). Graphs were drawn by Origin Software (version 2022, OriginLab Co., Northampton, MA, USA), and the significances were calculated by Student’s *t*-test.

The * represents *p* < 0.05, ** represents *p* < 0.01. (*n* ≥ 3).

## 3. Results

### 3.1. Characterization of Fluorinated PEG-PEI Coated MNPs

The elemental composition of F_7_-PEG-COOH was analyzed by SEM-EDS. The results are shown in Table S1. The atomic ratio of F, O, and C in F_7_-PEG-COOH is F:O:C = 1:7.288:15.25, which is basically consistent with the theoretical calculation of F:O:C =1:6.92:13.70. The FPP was dissolved in CD_3_ OD and characterized by ^19^ F-NMR, and the results are shown in Figure 2a. Compared with the published study [42], peak δ_1_ = −118.66 ppm is assigned to the fluorine atoms attached to the α carbon atom *(1)* of the heptafluorobutyryl. δ_2_ = −128.13 ppm is assigned to the fluorine atoms attached to the β carbon atom *(2)*. δ_3_ = −82.29 ppm is assigned to the fluorine atom attached to the γ carbon atom *(3)*. Hereby, it can be proved that the prepared product contains heptafluorobutyryl.

Magnetic nanoparticles (MNPs) were synthesized by pyrolysis of Fe(acac)_3_ at high temperatures and stored as a suspension in hexane. The TEM image of oleic acid-modified Fe_3_O_4_ magnetic nanoparticles (OA@MNPs) prepared by high-temperature pyrolysis is shown in Figure 2b. The size of MNPs is 11.92 ± 0.94 nm; the distribution of size is shown in Figure S1. After drying the OA@MNPs, the magnetization measured by the vibrating sample magnetometer (VSM) is shown in Figure 2h. The magnetization curves approximately pass through the (0,0) point, indicating that when the external magnetic field is 0, the magnetization of the MNPs is about 0. In addition, the maximum value indicates that the saturation magnetization of the OA@MNPs is 89.9 emu/g Fe (the Fe content of OA@MNPs was 70.2%). Figure 2k shows the high-resolution TEM (HRTEM) image of OA@MNPs. The lattice spacing (d) of MNPs is 0.295 nm, which is matched with the (220) plane of the cubic structure of Fe_3_O_4_. Figure 2l shows the selected area electron diffraction (SAED) of OA@MNPs. The electron diffraction rings are assigned to (111) (220) (311) (400) (422) (511) planes of the cubic structure of Fe_3_O_4_, respectively. The measurement of lattice spacing by HRTEM images is presented in Figure S2. The lattice spacings measured by HRTEM and SAED are shown in Table S2.

Then, using dimercaptosuccinic acid (DMSA), OA@MNPs were converted into water-soluble DMSA@MNPs and stored as a suspension in water. The TEM image of DMSA@MNPs is shown in Figure 2c, indicating that the morphology of MNPs remained after DMSA modification. F_7_-PEG-PEI coated MNPs (FPP@MNPs) were prepared by electrostatic adsorption. Figure 2d shows the TEM image of FPP@MNPs. It can be seen that the FPP-coated MNPs are dispersed instead of forming aggregates. The HAADF image of FPP@MNPs is shown in Figure 2e, and the distribution of F and Fe elements of FPP@MNPs is shown in Figure 2f,g. As can be seen, the distribution of F and Fe is highly correlated, indicating that FPP was combined with MNPs.

The hydrodynamic size and zeta potential of DMSA@MNPs and FPP@MNPs are shown in Figure 2i,j, which indicates that after F_7_-PEG-PEI modification, the size increased from 17.27 ± 0.32 nm to 93.29 ± 7.31 nm, and the zeta potential increased from −45.57 mV to 56.77 mV. A previous study reported that PEI-coated magnetic nanoparticles exhibited increased particle size and positive zeta potential [46]. The increase in size and the change of zeta potential from negative to positive illustrate that FPP is adsorbed on the surface of MNPs. Compared with the TEM images of FPP@MNPs and the hydrodynamic size of DMSA@MNPs, the hydrodynamic size of FPP@MNPs is significantly increased, suggesting that FPP@MNPs may aggregate in water. This phenomenon may be caused by the fluorophilic character between the fluorine chains.

### 3.2. Cell Viability and Apoptosis Assays

**Figure 3:**
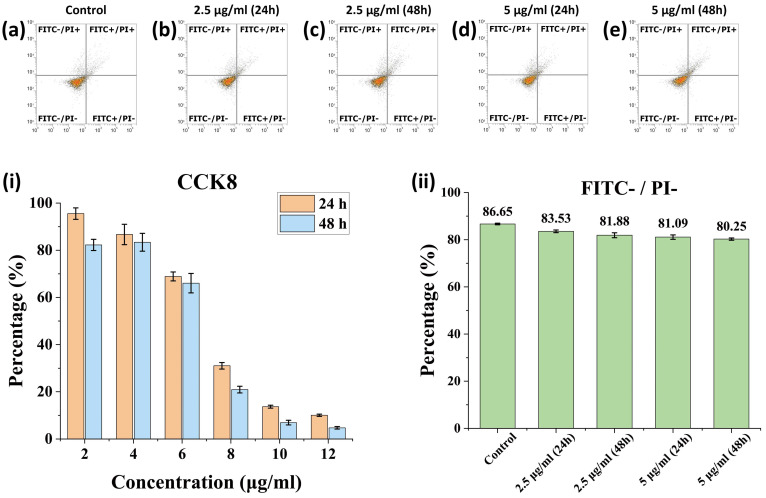
(**a**–**e**) Scatter diagram of HeLa cells apoptosis stained by Annexin V-FITC/PI measured by flow cytometry. (**i**) Relative cell viability measured by CCK8 kit. (**ii**) Analysis of cell viability measured by flow cytometry (**a**–**e**). The concentration is calculated by mass of Fe.

Figure 3i shows the effect of FPP@MNPs on cell viability, which was characterized by the CCK8 kit. Respectively, 2, 4, 6, 8, 10, and 12 μg/mL of FPP@MNP (calculated by the mass of Fe) were added to HeLa cells and then cultured for 24 h or 48 h. Compared with the cells incubating for 24 h, the viability of HeLa cells after 48 h of incubation was slightly reduced. When the concentration was lower than 4 μg/mL, FPP@MNPs showed low cytotoxicity (viability ≥ 80%). When the concentration reached 6 μg/mL, the cell viability decreased significantly. Furthermore, FPP@MNPs were toxic to HeLa cells when the concentration was ≥ 8 μg/mL. Meanwhile, the results of FPP@MNPs-induced HeLa cell apoptosis are shown in Figure 3a–e, and the analysis of cell viability is shown in Figure 3ii. In Figure 3a–e, the FITC-/PI- represents cells with normal viability. Figure 3ii shows that the inhibition of cell viability by FPP@MNPs with a concentration lower than 5 μg/mL was relatively slight, while the incubation time was not longer than 48 h.

Combining the results of CCK8 and apoptosis assays, it can be shown that when the concentration of FPP@MNPs is less than 4 μg/mL, the toxicity to cells is acceptable, which is much higher than that in our subsequent studies.

### 3.3. Validation of Cellular Uptake and Transfection Efficacy

**Figure 4:**
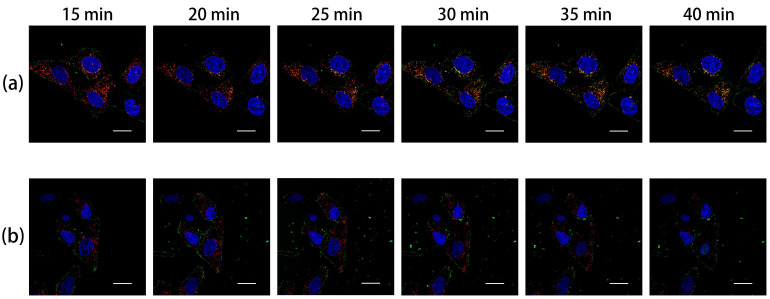
Uptake of (**a**) F_7_-PEG-PEI@MNP; (**b**) mPEG-PEI@MNP by HeLa cells. Figures were captured by confocal laser microscopy. Blue is Hoechst 33342 stained nuclei, Red is LysoTracker stained lysosomes, and Green is FITC-labeled nanoparticles. The result of co-localization of red and green fluorescence is yellow. (Scale = 20 μm).

Figure 4 shows the uptake of FITC-labeled F_7_-PEG-PEI@MNPs (FPP@MNPs) and mPEG-PEI@MNPs (mPP@MNPs) by HeLa cells. As shown in Figure 4a, 15 min after adding FPP@MNPs to HeLa cells, it was observed that particles (green fluorescence) were distributed around the cells and FPP@MNPs co-localized with lysosomes (red fluorescence). Moreover, from the beginning to 40 min, more co-localization can be observed. Prussian blue staining confirmed intracellular iron distribution (Figure S4).

As is shown in Figure 4b, from 15 min to 40 min after adding mPP@MNPs to HeLa cells, the nanoparticles around the cells could be observed. The intensity of green fluorescence gradually increased, indicating mPP@MNPs were gradually adsorbed on the cell membrane. In contrast, there is no observable co-localization of mPP@MNPs (green fluorescence) with lysosomes (red fluorescence) from the beginning until 40 min. Comparing Figure 4a,b, it is obvious that the cellular uptake efficiency of FPP-coated MNPs is significantly higher than mPP-coated MNPs.

Afterward, we used FPP@MNPs to load FAM-labeled siRNA (green fluorescence) and verified the transfection efficiency on human HeLa, A549 cell line, and mouse 4 T1 cell line. Cells were transfected at different FPP@MNPs/siRNA ratios and with or without external magnetic field enhancement. Specifically, the dosage of siRNA was 40 pmol, and the FPP@MNPs were 0.25, 0.5, 0.75, or 1.0 μg (mass of Fe). The method of external magnetic field enhancement is to place the cell culture plate on the magnetic plate for 15 min.

**Figure 5:**
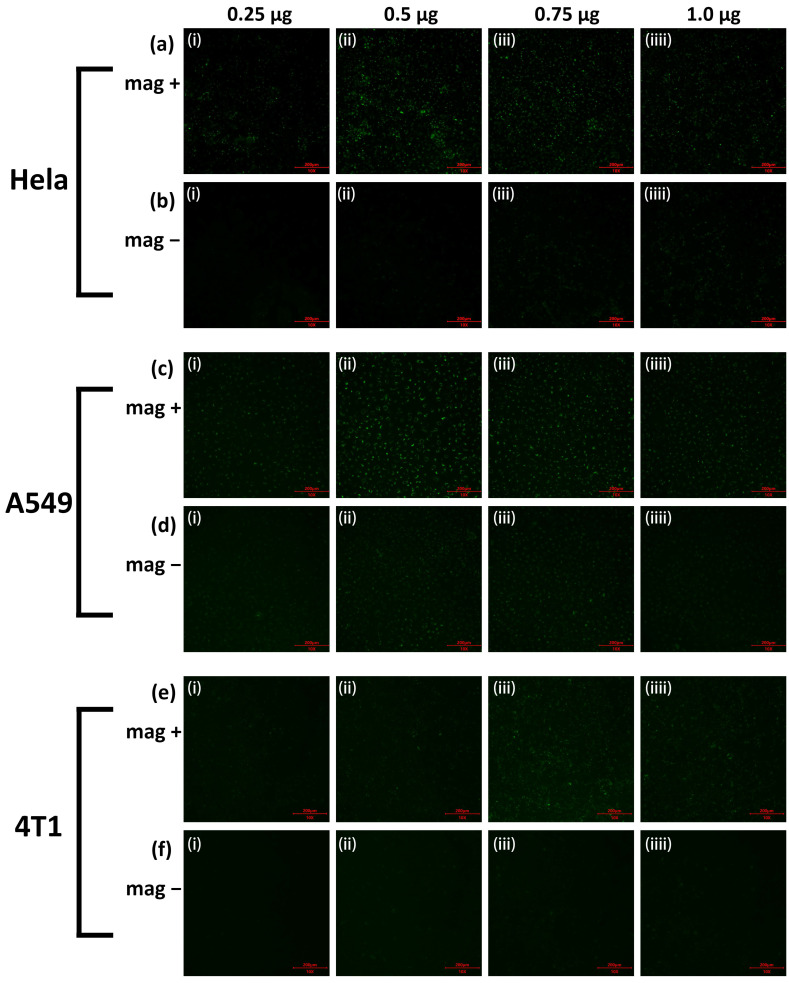
Fluorescence microscopic images of (**a**,**b**) HeLa, (**c**,**d**) A549, (**e**,**f**) 4 T1 cells transfected with FAM-siRNA. In total, 40 pmol FAM-siRNA mixed with 0.25 μg, 0.5 μg, 0.75 μg, or 1.0 μg (mass of Fe) F7-PEG-PEI@MNP, and then added to cells in 24-well plate. The effect of cell transfection enhanced by magnetic fields (mag+) or not (mag−). Excitation λ = 488 nm. (Scale = 200 μm).

Figure 5 shows the images taken by fluorescence microscopy. After 12 h, the results of each group show that the efficiency of siRNA delivery by FPP@MNPs can be significantly enhanced by an external magnetic field (mag+) compared to the transfections without magnetic field enhancement (mag−). For HeLa cells and A549 cells, 0.5 μg or 0.75 μg of FPP@MNPs combined with 40 pmol of siRNA-NC can achieve high transfection efficiency by the magnetic field enhancement. For 4 T1 cells, 0.75 μg of FPP@MNPs combined with 40 pmol of siRNA NCs can achieve high-efficiency transfection under the enhancement of the magnetic field. Overall, 0.75 μg FPP@MNPs with 40 pmol of siRNA are preferred.

**Figure 6:**
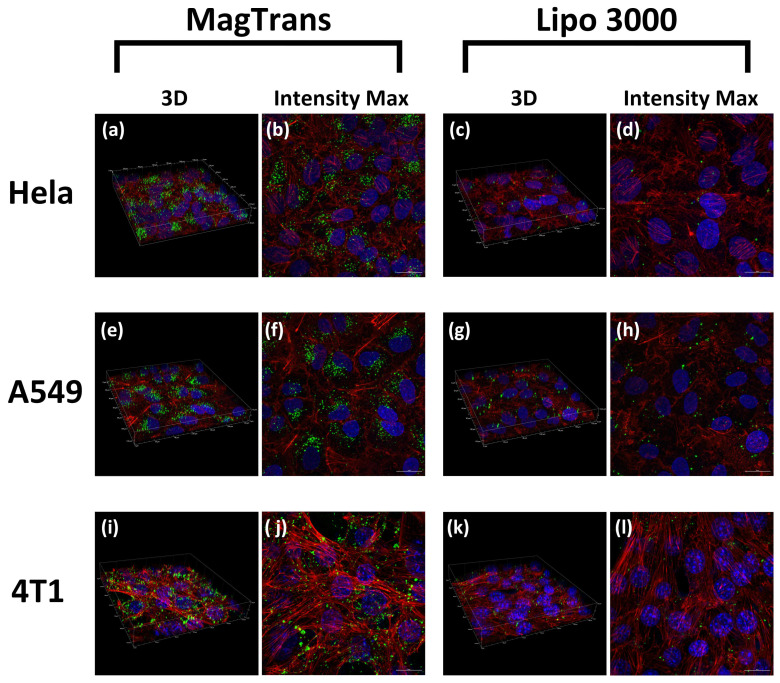
Results of FAM-siRNA transfection captured by confocal microscopy. Blue is Hoechst33342 stained nuclei, Green is FAM-labeled siRNA NC, and Red is Rhodamine-Phalloidin stained F-actin (**a**–**d**) HeLa, (**e**–**h**) A549, (**i**–**l**) 4 T1. Transfection method used were magnetic field-enhanced F_7_-PEG-PEI@MNP transfection (MagTrans) or Lipo3000 transfection (Lipo 3000). Images are reconstructed in 3 D, and the images with the highest fluorescence intensity (Intensity Max) are given by NIS-Elements software (Nikon Co., Tokyo, Japan).

As is shown in Figure 6, in order to further explore the distribution of siRNA(FAM-labeled, green fluorescence) in cells, the cells were scanned layer by layer and three-dimensionally reconstructed by laser confocal microscopy. HeLa, A549, and 4 T1 cells were transfected with FPP@MNPs under external magnetic field enhancement (MagTrans). The commercial nucleic acid vector Invitrogen Lipofectamine 3000 Reagent (Lipo3000) was used as the control. For each group of cells, it is evident that the amount of siRNA inside MagTrans treated cells is significantly higher than Lipo3000 transfected cells. For Lipo3000 transfected cells, the distribution of siRNA can be observed in almost every cell, but the amount of siRNA contained in a single cell is far less than that of cells treated by MagTrans.

**Figure 7:**
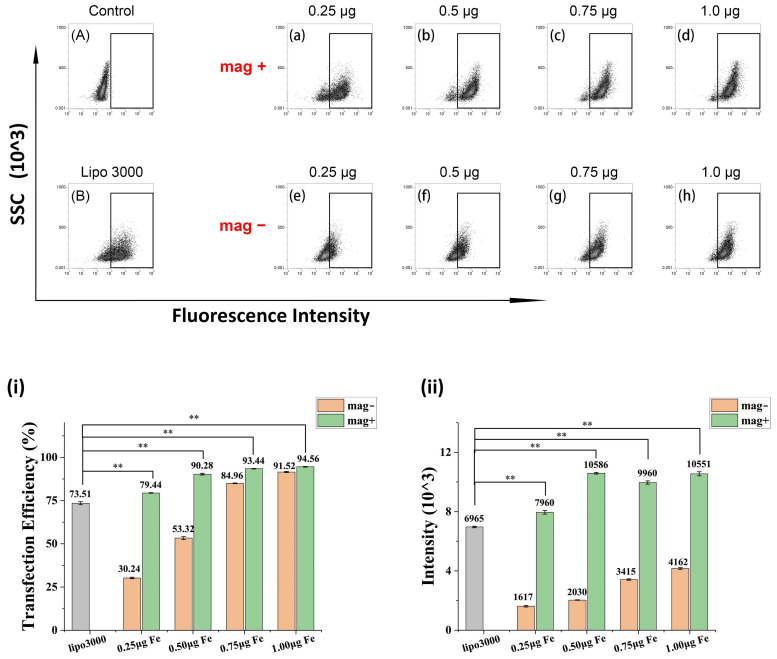
Transfection efficiency of HeLa cells measured by flow cytometry. (**A**) Control. (**B**) Lipo 3000 transfection. (**a**–**d**) 40 pmol FAM-siRNA mixed with 0.25 μg, 0.5 μg, 0.75 μg, or 1.0 μg F_7_-PEG-PEI@MNP, transfection enhanced by magnetic fields (mag+). (**e**–**h**) 40 pmol FAM-siRNA mixed with F_7_-PEG-PEI@MNP (mag-). (**i**) Transfection efficiency of Lipo3000, mag+ and mag-. **(ii)** Mean fluorescence intensity of Lipo3000, mag+ and mag− transfected cells (gated). ** *p* < 0.01.

The transfection efficiency of HeLa cells was analyzed by flow cytometry, and the results are shown in Figure 7. The dots inside the rectangular gate indicate transfected cells. Figure 7A shows untreated HeLa cells as a negative control, and Figure 7B shows HeLa cells transfected with Lipo3000 as the positive control. The results of magnetic field enhanced FPP@MNPs transfection (mag+) are shown in Figure 7a–d, and the results of FPP@MNPs transfection without magnetic-field enhancement (mag−) are shown in Figure 7e–h. The statistical analysis of transfection efficiency and mean fluorescence intensity of each group is shown in Figure 7i,ii.

In Figure 7i, comparing the Mag+ group with the Mag- group, the external magnetic field could effectively improve the transfection efficiency. In particular, the improvement was more pronounced between the groups that used fewer FPP@MNPs (0.25 μg or 0.5 μg, mass of Fe, the same below). Furthermore, the difference in transfection efficiency between the mag+ group and Lipo3000 is significant. The transfection efficiency of Lipo3000 was 73.51%, and the transfection efficiency of 0.25 μg FPP@MNP was 79.44%, which was slightly improved. For the transfection results of 0.5 μg, 0.75 μg, and 1.0 μg FPP@MNP, the efficiency was higher than 90%, which was greatly improved compared with Lipo3000.

Figure 7ii shows the statistical analysis of the Mean Fluorescence Intensity (MFI) of transfected cells (within the rectangular gate). Compared with the mag+ group and the mag - group, the magnetic field-enhanced transfection could not only improve the transfection efficiency but also increase the content of siRNA transfected into cells. Comparing the mag+ group with the Lipo3000 group, it can be seen that the MFI of the 0.25 μg mag+ was slightly higher than that of the Lipo3000. The MFI of 0.5 μg, 0.75 μg, and 1.0 μg FPP@MNP transfection was much higher than that of the Lipo3000, and the difference was significant.

### 3.4. Knockdown of CXCR4 Expression in 4 T1 Cells

**Figure 8:**
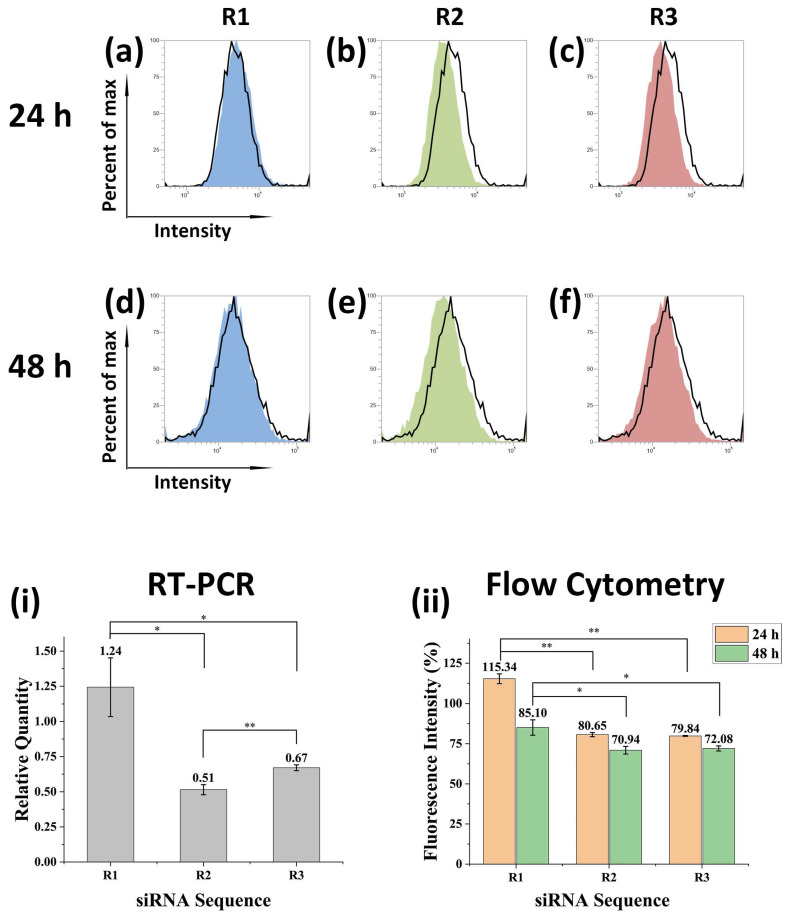
Results of CXCR4 expression and knockdown on 4 T1 cells. (**a**–**c**) 24 h after transfection, fluorescence intensity of Cy5. (**d**–**f**) 48 h after transfection, fluorescence intensity of Cy5. CXCR4 were detected by Cy5 labelled antibody, peaks with black lines represent negative control. (**i**) Analysis of CXCR4 mRNA measured by RT-PCR. (**ii**) Analysis of the relative intensity of CXCR4 expression on cell membranes measured by flow cytometry (**a**–**f**). * *p* < 0.05, ** *p* < 0.01.

Then, siRNA sequences (R1-R3) for silencing CXCR4 (siCXCR4) were designed and synthesized. The sequences of negative control (NC) and siCXCR4 are given in Table 2. For 4 T1 cells in the 24-well cell culture plate, 0.75 μg of FPP@MNP and 40 pmol of siRNA were mixed for transfection, which was enhanced with an external magnetic field. Cells were cultured for 12 h after transfection, and CXCR4 mRNA was detected by RT-PCR. The results are shown in Figure 8i. With the CXCR4 mRNA in the NC group as a reference, the relative expression levels of the R1, R2, and R3 groups were calculated after normalization. As can be seen in Figure 8i, the knockdown rate of R1 is not ideal and may even lead to increased CXCR4 expression. R2 and R3 can effectively reduce CXCR4 mRNA, and R2 achieves the best effect. The above differences are all significant.

The CXCR4 expression on the membrane of 4 T1 cells after transfection for 24 h or 48 h was detected by flow cytometry. The fluorescence intensity distribution of Cy5 labeled anti-CXCR4 antibody is shown in Figure 8a–f, and the analysis is shown in Figure 8ii. After 24 h, the expression of CXCR4 in the R2 and R3 groups decreased, while the expression of CXCR4 in the R1 group increased compared with the negative control. The difference between R1 and R2, R1 and R3 was extremely significant, but the difference between R2 and R3 was not significant. After 48 h, the expression of CXCR4 in the R1, R2, and R3 groups was decreased compared with the cells transfected for 24 h. However, the knockdown efficiency of R1 was still lower than that of R2 or R3, and the difference was significant. Besides, the difference between R2 and R3 is still not significant.

To sum up, the CXCR4 knockdown rate increased as the culture time of the transfected cells increased from 24 h to 48 h. Compared to the efficiencies of the three sequences (R1, R2, and R3), the effect of R1 was poor. Even R1 may cause an increase in CXCR4 expression for a short period of time and should therefore be excluded. At the protein level, the effect of R2 and R3 was not significantly different. However, at the mRNA level, the knockdown effect of R2 was extremely significant compared to R3.

## 4. Discussion

The preparation process of F_7_-PEG-PEI (FPP) includes the preparation of F_7_-PEG-COOH and the reaction of F_7_-PEG-COOH with polyethyleneimine (PEI). The measurement of the three elements (C, O, and F) of F_7_-PEG-COOH shows that the proportion of the F element is 4.23%. Theoretically, when a heptafluorobutyryl group is attached to a PEG_2000_ molecule, the proportion of the F element is 4.63%. The ratio of the actual value to the theoretical value was 4.23/4.63 = 91.36%, which indicated that most of the PEG molecules were modified with heptafluorobutyryl. The EDC/NHS system is widely used for activating carboxyl groups. The carboxyl group of F_7_-PEG-COOH was activated by EDC/NHS and then reacted with PEI to synthesize FPP. The ^19^ F-NMR results of FPP proved the existence of heptafluorobutyryl.

Nowadays, MNPs are widely used in biomedical fields such as MRI, drug delivery, and hyperthermia [47]. Compared with the magnetic nanoparticles prepared by co-precipitation, the size distribution of the MNPs prepared by high-temperature pyrolysis is more uniform, and the saturation magnetization is higher [48]. Magnetite nanoparticles smaller than 25 nm in size are considered to be superparamagnetic, which is defined as the magnetic material that has no magnetization in the absence of an external magnetic field [21]. The TEM size of prepared Fe_3_O_4_ nanoparticles is 11.92 ± 0.94 nm, and the magnetization curve in Figure 2h proves that our prepared Fe_3_O_4_ nanoparticles are superparamagnetic. The increase in hydrodynamic size and the change of zeta potential demonstrated that FPP was coated on the surface of DMSA@MNPs, and the EDS mapping confirmed that the nanoparticles were fluorinated. In our work, the results of SAED are in good agreement with previous studies [49,50], which is consistent with the inverse spinel structure of Fe_3_O_4_. Besides, the lattice spacing of the (220) plane measured by HRTEM is 0.295 nm, which corroborates with the 0.298 nm referred to in a previous study [50].

As shown in the introduction part [42,43,44,45], it is shown in many studies that fluorination can improve the transfection efficiency of polymer nanoparticles. Different from previous studies, we modified PEG with heptafluorobutyric anhydride and then connected it with PEI. Using fluorinated PEG-PEI to coat MNPs can not only enhance cellular uptake by fluorination but also enhance transfection efficiency by a magnetic field. Compared with the commercial vector Lipo3000, our prepared FPP@MNPs can transfect more siRNA into cells. As can be seen from Figure 6, Lipo3000 has high transfection efficiency, but the amount of siRNA delivered into cells is not as high as that of FPP@MNPs (enhanced by magnetic field). These claims can also be confirmed by the results of flow cytometry in Figure 7.

Previous studies have established that CXCR4 is required for breast cancer cell proliferation or survival, and CXCR4 inhibitors will improve the treatment of primary and metastatic breast cancer [51,52]. The high expression of CXCR4 in 4 T1 breast cancer cells was widely demonstrated in much research [53,54,55]. From the previous studies, this mouse cell line is suitable for breast cancer research, including in vitro studies or the construction of mouse models for in vivo studies. We used the FPP@MNPs prepared in this study as siRNA carriers to transfect 4 T1 cells and knock down the expression of CXCR4 in vitro. The effect of FPP@MNPs as siRNA carriers has been confirmed, and our research on HeLa cells and A549 cells shows that FPP@MNPs can be extended to more applications.

## 5. Conclusions

Based on magnetic nanoparticles, fluorinated siRNA carriers F_7_-PEG-PEI@MNPs were developed in this study. The carriers were less toxic to cells at conventional doses and allowed for efficient cellular uptake. Our findings suggest that fluorination is an effective strategy to overcome the “PEG dilemma”. The results of transfection showed that our prepared FPP@MNPs could achieve an efficiency of more than 90%. In particular, the transfection efficiency can be further improved by an external magnetic field, even with a smaller amount of FPP@MNPs. In vitro experiments confirmed that FPP@MNPs could be used to knockdown CXCR4 expression in breast cancer cells. Furthermore, this vector was verified to be universal on different cells.

## 6. Patents

Heptafluorobutyryl-polyethylene glycol-polyethyleneimine (F_7_-PEG-PEI) and nanoparticles prepared with F_7_-PEG-PEI and the above technical solutions and applications have been submitted for patent examination in the People’s Republic of China. All rights are hereby declared. Application Number [202210144467.8].

## Figures and Tables

**Table 1:** Sequences of primer.

Target (Mouse)	Primer	
Actin	F	CTCCTGAGCGCAAGTACTCT
	R	TACTCCTGCTTGCTGATCCAC
CXCR4	F	TTCATCTTTGCCGACGTCAG
	R	CGAGACCCACCATTATATGCT

**Table 2:** siRNA Sequences.

siRNA	Sequence
NC	(5′-3′) UUCUCCGAACGUGUCACGUTT (3′-5′) TTAAGAGGCUUGCACAGUGCA
R1	(5′-3′) CGAUCAGUGUGAGUAUAUATT (3′-5′) TTGCUAGUCACACUCAUAUAU
R2	(5′-3′) GUCCAUUUCAAUAGGAUCUTT (3′-5′) TTCGGAGUUCUAGGAAAGGUU
R3	(5′-3′) GCCUCAAGAUCCUUUCCAATT (3′-5′) TTCGGAGUUCUAGGAAAGGUU

## Data Availability

Not applicable.

## Supplementary Materials

The following supporting information can be downloaded at: https://www.mdpi.com/2079-4991/12/10/1692/s1, Table S1: Relative Content of C, O, F Elements in F7-PEG-COOH measured by EDS; Table S2: Lattice spacing measured by HRTEM and SAED; Figure S1: TEM size distribution of MNPs; Figure S2: Filtered HRTEM image for measuring lattice spacing of Fe_3_O_4_ NPs; Figure S3: Standard curve for measuring Fe concentration; Figure S4. Fe^3+^ distribution in Hela cells measured by Prussian blue Staining; Figure S5. DLS distribution of mPEG-PEI@MNPs; Method S1: Preparation of mPEG-PEI@MNPs (mPP@MNPs); Method S2. Measurement of Fe concentration by UV-Vis Spectrophotometry; Method S3. Prussian blue staining.

## References

[1] Li Z., Wang Y., Shen Y., Qian C., Oupicky D., Sun M. (2020). Targeting pulmonary tumor microenvironment with CXCR4-inhibiting nanocomplex to enhance anti–PD-L1 immunotherapy. Sci. Adv..

[2] Arya M., Patel H.R.H., McGurk C., Tatoud R., Klocker H., Masters J., Williamson M. (2004). The Importance of the CXCL12-CXCR4 chemokine ligand-receptor interaction in prostate cancer metastasis. J. Exp. Ther. Oncol..

[3] Liang Z., Yoon Y., Votaw J., Goodman M.M., Williams L., Shim H. (2005). Silencing of CXCR4 blocks breast cancer metastasis. Cancer Res..

[4] Xue S., Ma M., Bei S., Li F., Wu C., Li H., Hu Y., Zhang X., Qian Y., Qin Z. (2021). Identification and Validation of the Immune Regulator CXCR4 as a Novel Promising Target for Gastric Cancer. Front. Immunol..

[5] Passaro D., Irigoyen M., Catherinet C., Gachet S., Da Costa De Jesus C., Lasgi C., Tran Quang C., Ghysdael J. (2015). CXCR4 is required for leukemia-initiating cell activity in T cell acute lymphoblastic leukemia. Cancer Cell.

[6] Tseng D., Vasquez-Medrano D.A., Brown J.M. (2011). Targeting SDF-1/CXCR4 to inhibit tumour vasculature for treatment of glioblastomas. Br. J. Cancer.

[7] Müller A., Homey B., Soto H., Ge N., Catron D., Buchanan M.E., McClanahan T., Murphy E., Yuan W., Wagner S.N. (2001). Involvement of chemokine receptors in breast cancer metastasis. Nature.

[8] Giallongo C., Dulcamare I., Tibullo D., del Fabro V., Vicario N., Parrinello N., Romano A., Scandura G., Lazzarino G., Conticello C. (2022). CXCL12/CXCR4 axis supports mitochondrial trafficking in tumor myeloma microenvironment. Oncogenesis.

[9] Meng J., Ge Y., Xing H., Wei H., Xu S., Liu J., Yan D., Wen T., Wang M., Fang X. (2020). Synthetic CXCR4 antagonistic peptide assembling with nanoscaled micelles combat acute myeloid leukemia. Small.

[10] Donzella G.A., Schols D., Lin S.W., Esté J.A., Nagashima K.A., Maddon P.J., Allaway G.P., Sakmar T.P., Henson G., De Clercq E. (1998). AMD3100, a small molecule inhibitor of HIV-1 Entry via the CXCR4 Co-Receptor. Nat. Med..

[11] Tamamura H., Hiramatsu K., Kusano S., Terakubo S., Yamamoto N., Trent J.O., Wang Z., Peiper S.C., Nakashima H., Otaka A. (2003). Synthesis of Potent CXCR4 inhibitors possessing low cytotoxicity and improved biostability based on T140 derivatives. Organic Biomol. Chem..

[12] Li X., Guo H., Yang Y., Meng J., Liu J., Wang C., Xu H. (2015). A designed peptide targeting CXCR4 displays anti-acute myelocytic leukemia activity in vitro and in vivo. Sci. Rep..

[13] Lam J.K.W., Chow M.Y.T., Zhang Y., Leung S.W.S. (2015). SiRNA versus MiRNA as therapeutics for gene silencing. Mol. Ther. Nucl. Acids.

[14] Elbashir S.M., Harborth J., Lendeckel W., Yalcin A., Weber K., Tuschl T. (2001). Duplexes of 21-Nucleotide RNAs Mediate RNA interference in cultured mammalian cells. Nature.

[15] Setten R.L., Rossi J.J., Han S. (2019). The current state and future directions of RNAi-based therapeutics. Nat. Rev. Drug Discov..

[16] Lim L.P., Lau N.C., Garrett-Engele P., Grimson A., Schelter J.M., Castle J., Bartel D.P., Linsley P.S., Johnson J.M. (2005). Microarray analysis shows that some MicroRNAs downregulate large numbers of target MRNAs. Nature.

[17] Landry B., Gül-Uludağ H., Plianwong S., Kucharski C., Zak Z., Parmar M.B., Kutsch O., Jiang H., Brandwein J., Uludağ H. (2016). Targeting CXCR4/SDF-1 axis by lipopolymer complexes of SiRNA in acute myeloid leukemia. J. Control. Release.

[18] Mykhaylyk O., Vlaskou D., Tresilwised N., Pithayanukul P., Möller W., Plank C. (2007). Magnetic nanoparticle formulations for DNA and SiRNA delivery. J. Magn. Magn. Mater..

[19] Liu D., Cheng Y., Cai R., Wang B.W., Cui H., Liu M., Zhang B., Mei Q., Zhou S. (2018). The enhancement of SiPLK1 penetration across BBB and its anti glioblastoma activity in vivo by magnet and transferrin co-modified nanoparticle. Nanomed. Nanotechnol. Biol. Med..

[20] Babu A., Wang Q., Muralidharan R., Shanker M., Munshi A., Ramesh R. (2014). Chitosan coated polylactic acid nanoparticle-mediated combinatorial delivery of cisplatin and SiRNA/Plasmid DNA chemosensitizes cisplatin-resistant human ovarian cancer cells. Mol. Pharm..

[21] Estelrich J., Escribano E., Queralt J., Busquets M. (2015). Iron oxide nanoparticles for magnetically-guided and magnetically-responsive drug delivery. Int. J. Mol. Sci..

[22] Hoang Thi T.T., Pilkington E.H., Nguyen D.H., Lee J.S., Park K.D., Truong N.P. (2020). The importance of poly(ethylene glycol) alternatives for overcoming PEG immunogenicity in drug delivery and bioconjugation. Polymers.

[23] Koren E., Apte A., Jani A., Torchilin V.P. (2012). Multifunctional PEGylated 2C5-immunoliposomes containing PH-sensitive bonds and TAT peptide for enhanced tumor cell internalization and cytotoxicity. J. Control. Release.

[24] Mohamed M., Abu Lila A.S., Shimizu T., Alaaeldin E., Hussein A., Sarhan H.A., Szebeni J., Ishida T. (2019). PEGylated Liposomes: Immunological responses. Sci. Technol. Adv. Mater..

[25] Zhang W., Guo Z., Huang D., Liu Z., Guo X., Zhong H. (2011). Synergistic effect of chemo-photothermal therapy using PEGylated graphene oxide. Biomaterials.

[26] Chen M., Tang S., Guo Z., Wang X., Mo S., Huang X., Liu G., Zheng N. (2014). Core-Shell Pd@Au nanoplates as theranostic agents for in-vivo photoacoustic imaging, CT imaging, and photothermal therapy. Adv. Mater..

[27] Wang M., Chang M., Chen Q., Wang D., Li C., Hou Z., Lin J., Jin D., Xing B. (2020). Au2Pt-PEG-Ce6 nanoformulation with dual nanozyme activities for synergistic chemodynamic therapy/phototherapy. Biomaterials.

[28] Ciocci M., Cacciotti I., Seliktar D., Melino S. (2018). Injectable silk fibroin hydrogels functionalized with microspheres as adult stem cells-carrier systems. Int. J. Biol. Macromol..

[29] Masood N., Ahmed R., Tariq M., Ahmed Z., Masoud M.S., Ali I., Asghar R., Andleeb A., Hasan A. (2019). Silver nanoparticle impregnated chitosan-PEG hydrogel enhances wound healing in diabetes induced rabbits. Int. J. Pharm..

[30] Grun M.K., Suberi A., Shin K., Lee T., Gomerdinger V., Moscato Z.M., Piotrowski-Daspit A.S., Saltzman W.M. (2021). PEGylation of poly(amine-co-ester) polyplexes for tunable gene delivery. Biomaterials.

[31] Hoskins C., Wang L., Cheng W.P., Cuschieri A. (2012). Dilemmas in the reliable estimation of the in-vitro cell viability in magnetic nanoparticle engineering: Which tests and what protocols?. Nanoscale Res. Lett..

[32] Sun C.-Y., Shen S., Xu C.-F., Li H.-J., Liu Y., Cao Z.-T., Yang X.-Z., Xia J.-X., Wang J. (2015). Tumor Acidity-Sensitive Polymeric Vector for Active Targeted SiRNA Delivery. J. Am. Chem. Soc..

[33] Fang Y., Xue J., Gao S., Lu A., Yang D., Jiang H., He Y., Shi K. (2017). Cleavable PEGylation: A strategy for overcoming the “PEG Dilemma” in efficient drug delivery. Drug Deliv..

[34] Chen Y., Zhang M., Jin H., Tang Y., Wang H., Xu Q., Li Y., Li F., Huang Y. (2017). Intein-mediated site-specific synthesis of tumor-targeting protein delivery system: Turning PEG dilemma into prodrug-like feature. Biomaterials.

[35] Kanamala M., Palmer B.D., Jamieson S.M., Wilson W.R., Wu Z. (2019). Dual PH-sensitive liposomes with low PH-triggered sheddable PEG for enhanced tumor-targeted drug delivery. Nanomedicine.

[36] Han X., Li Y., Xu Y., Zhao X., Zhang Y., Yang X., Wang Y., Zhao R., Anderson G.J., Zhao Y. (2018). Reversal of pancreatic desmoplasia by re-educating stellate cells with a tumour microenvironment-activated nanosystem. Nat. Commun..

[37] Zhu Y., Chen C., Cao Z., Shen S., Li L., Li D., Wang J., Yang X. (2019). On-demand PEGylation and DePEGylation of PLA-Based nanocarriers via amphiphilic MPEG-*TK*-Ce6 for nanoenabled cancer chemotherapy. Theranostics.

[38] Fan G., Fan M., Wang Q., Jiang J., Wan Y., Gong T., Zhang Z., Sun X. (2016). Bio-inspired polymer envelopes around adenoviral vectors to reduce immunogenicity and improve in vivo kinetics. Acta Biomater..

[39] Li G., Song Y., Huang Z., Chen K., Chen D., Deng Y. (2017). Novel, nano-sized, liposome-encapsulated polyamidoamine dendrimer derivatives facilitate tumour targeting by overcoming the polyethylene glycol dilemma and integrin saturation obstacle. J. Drug Target..

[40] Hou M., Wu X., Zhao Z., Deng Q., Chen Y., Yin L. (2022). Endothelial cell-targeting, ROS-Ultrasensitive Drug/SiRNA co-delivery nanocomplexes mitigate early-stage neutrophil recruitment for the anti-inflammatory treatment of myocardial ischemia reperfusion injury. Acta Biomater..

[41] Shuai Q., Cai Y., Zhao G., Sun X. (2020). Cell-penetrating peptide modified PEG-PLA micelles for efficient PTX Delivery. Int. J. Mol. Sci..

[42] Lv J., Chang H., Wang Y., Wang M., Xiao J., Zhang Q., Cheng Y. (2015). Fluorination on polyethylenimine allows efficient 2D and 3D cell culture gene delivery. J. Mater. Chem. B.

[43] Wang H., Wang Y., Wang Y., Hu J., Li T., Liu H., Zhang Q., Cheng Y. (2015). Self-assembled fluorodendrimers combine the features of lipid and polymeric vectors in gene delivery. Angew. Chem. Int. Ed..

[44] Wang M., Liu H., Li L., Cheng Y. (2014). A fluorinated dendrimer achieves excellent gene transfection efficacy at extremely low nitrogen to phosphorus ratios. Nat. Commun..

[45] Chen G., Wang Y., Ullah A., Huai Y., Xu Y. (2020). The effects of fluoroalkyl chain length and density on sirna delivery of bioreducible poly(amido amine)s. Eur. J. Pharm. Sci..

[46] Zhang L., Li Y., Yu J.C., Chen Y.Y., Chan K.M. (2014). Assembly of polyethylenimine-functionalized iron oxide nanoparticles as agents for DNA transfection with magnetofection technique. J. Mater. Chem. B.

[47] Obaidat I., Issa B., Haik Y. (2015). Magnetic properties of magnetic nanoparticles for efficient hyperthermia. Nanomaterials.

[48] Sun S., Zeng H., Robinson D.B., Raoux S., Rice P.M., Wang S.X., Li G. (2004). Monodisperse MFe_2_O_4_ (M = Fe, Co, Mn) Nanoparticles. J. Am. Chem. Soc..

[49] Ghosh R., Pradhan L., Devi Y.P., Meena S.S., Tewari R., Kumar A., Sharma S., Gajbhiye N.S., Vatsa R.K., Pandey B.N. (2011). Induction Heating Studies of Fe_3_O_4_ magnetic nanoparticles capped with oleic acid and polyethylene glycol for hyperthermia. J. Mater. Chem..

[50] Wan Q., Xie L., Gao L., Wang Z., Nan X., Lei H., Long X., Chen Z.-Y., He C.-Y., Liu G. (2013). Self-assembled magnetic theranostic nanoparticles for highly sensitive MRI of minicircle DNA delivery. Nanoscale.

[51] Smith M.C.P., Luker K.E., Garbow J.R., Prior J.L., Jackson E., Piwnica-Worms D., Luker G.D. (2004). CXCR4 regulates growth of both primary and metastatic breast cancer. Cancer Res..

[52] Wang Z., Ma Y., Yu X., Niu Q., Han Z., Wang H., Li T., Fu D., Achilefu S., Qian Z. (2018). Targeting CXCR4-CXCL12 Axis for visualizing, predicting, and inhibiting breast cancer metastasis with theranostic AMD3100-Ag_2_S Quantum Dot Probe. Adv. Funct. Mater..

[53] Zhang F., Gong S., Wu J., Li H., Oupicky D., Sun M. (2017). CXCR4-targeted and redox responsive dextrin nanogel for metastatic breast cancer therapy. Biomacromolecules.

[54] Li H., Zhang X., Wu H.Y., Sun L., Ma Y., Xu J., Lin Q., Zeng D. (2019). ^64^ Cu-labeled ubiquitin for PET imaging of CXCR4 expression in mouse breast tumor. ACS Omega.

[55] Mikaeili A., Erfani M., Goudarzi M., Sabzevari O. (2021). Breast tumor targeting in mice bearing 4T1 tumor with labeled CXCR4 antagonist analogue. Int. J. Peptide Res. Ther..

