# Correction: Cao et al. Fluorinated PEG-PEI Coated Magnetic Nanoparticles for siRNA Delivery and CXCR4 Knockdown. *Nanomaterials* 2022, *12*, 1692

**DOI:** 10.3390/nano14100830

**Published:** 2024-05-09

**Authors:** Yixiang Cao, Shiyin Zhang, Ming Ma, Yu Zhang

**Affiliations:** 1 State Key Laboratory of Bioelectronics, Jiangsu Key Laboratory for Biomaterials and Devices, School of Biological Sciences and Medical Engineering, Southeast University, Nanjing 210096, China; 220195143@seu.edu.cn; 2 Nanjing Nanoeast Biotech Co., Ltd., Nanjing 211000, China; syzhang@nanoeast.net

## Citation Correction

In the original publication [1], Xue, S.; Ma, M.; Bei, S.; Li, F.; Wu, C.; Li, H.; Hu, Y.; Zhang, X.; Qian, Y.; Qin, Z.; et al. Identification and Validation of the Immune Regulator CXCR4 as a Novel Promising Target for Gastric Cancer. *Front. Immunol.* **2021**, *12*, 702615. https://doi.org/10.3389/fimmu.2021.702615 was not cited. The citation has now been inserted in Section 1. Introduction, Paragraph 1, and has replaced the original citation (ref. 4), Han, M.; Lv, S.; Zhang, Y.; Yi, R.; Huang, B.; Fu, H.; Bian, R.; Li, X. The prognosis and clinicopathology of CXCR4 in gastric cancer patients: A meta-analysis. *Tumor Biol.* **2014**, *35*, 4589–4597.

The authors state that the scientific conclusions are unaffected. This correction was approved by the Academic Editor. The original publication has also been updated.

## References

[1] Cao Y., Zhang S., Ma M., Zhang Y. (2022). Fluorinated PEG-PEI Coated Magnetic Nanoparticles for siRNA Delivery and CXCR4 Knockdown. Nanomaterials.

