# Supplementary material for: Fluorinated PEG-PEI Coated Magnetic Nanoparticles for siRNA Delivery and CXCR4 Knockdown
Source: Nanomaterials (Basel). 2022 May 16;12(10):1692. doi: 10.3390/nano12101692 (PMC9146302; doi:10.3390/nano12101692)
Supplement: Supplementary file 1 [file nanomaterials-12-01692-s001.zip › nanomaterials-1707003-supplementary.pdf]

Supplementary Information

Fluorinated PEG-PEI Coated Magnetic Nanoparticles for siRNA Delivery and CXCR4 Knockdown

Yixiang Cao [1], Shiyin Zhang [2], Ming Ma [1,*] and Yu Zhang [1,*]

[1] State Key Laboratory of Bioelectronics, Jiangsu Key Laboratory for Biomaterials and Devices,
School of Biological Sciences and Medical Engineering, Southeast University, Nanjing 210096, China; 220195143@seu.edu.cn

[2] Nanjing Nanoeast Biotech Co., Ltd., Nanjing 211000, China; syzhang@nanoeast.net

* Correspondence: maming@seu.edu.cn (M.M.); zhangyu@seu.edu.cn (Y.Z.)

Supplementary Tables and Figures

Table S1. Relative Content of C, O, F Elements in F_7-PEG-COOH measured by EDS

Elements	Percentage of weight	Percentage of atoms
C	57.62%	64.94%
O	36.44%	30.83%
F	5.94%	4.23%

Table S2. Lattice spacing measured by HRTEM and SAED. The results were compared with the Standard lattice spacing of each plane (According to MDI Jade)

**Planes** (Fe_3O_4)	***Standard*** *d(nm)*	**SAED** d(nm)	**HRTEM** d(nm)
(111)	*0.467*	0.439	
(220)	*0.286*	0.297	0.295
(311)	*0.244*	0.254	
(222)	*0.234*		
(400)	*0.202*	0.215	
(422)	*0.165*	0.168	
(511)	*0.156*	0.154	
(440)	*0.143*		
(533)	*0.123*		

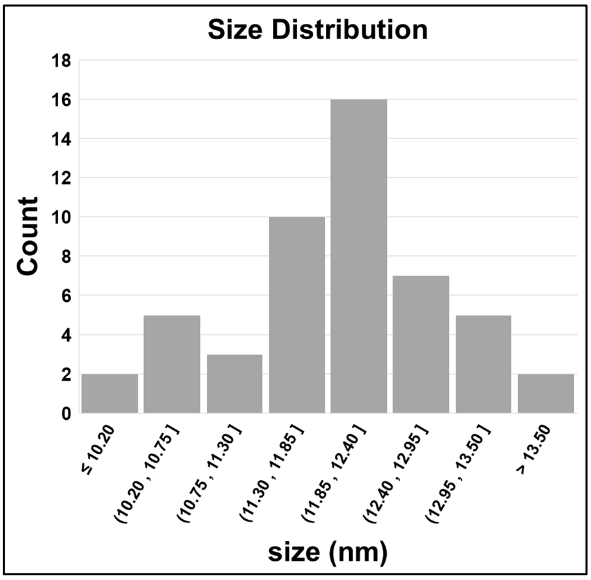

Figure S1. TEM size distribution of MNPs.
The mean size of prepared Fe_3O_4 nanoparticles is 11.92 ± 0.94 nm

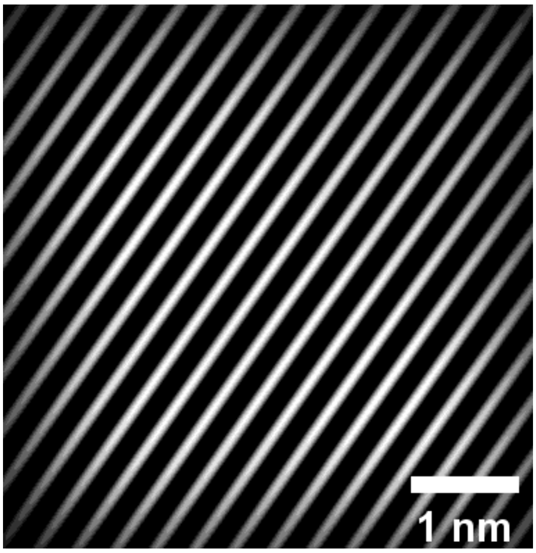

Figure S2. Filtered HRTEM image for measuring lattice spacing of Fe_3O_4 NPs

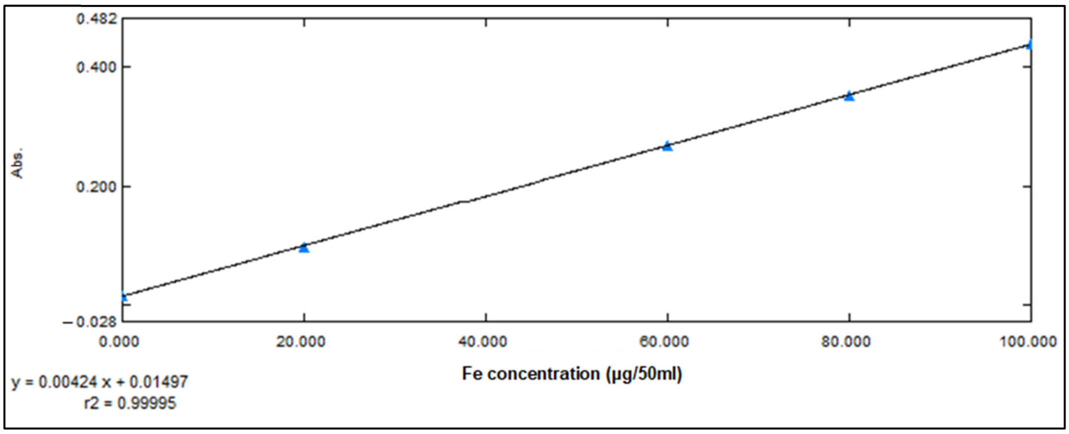

Figure S3. Standard curve for measuring Fe concentration

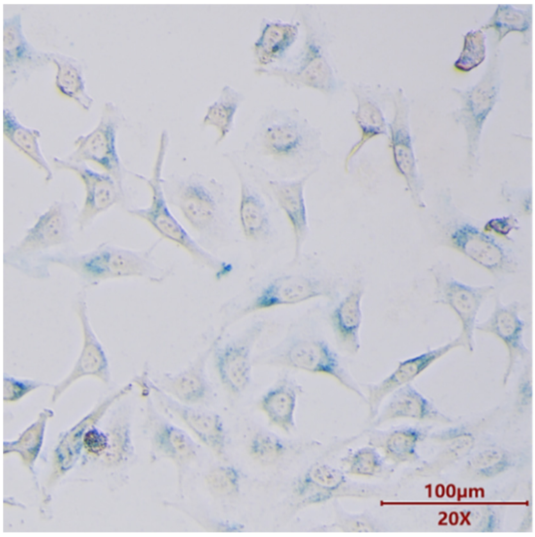

Figure S4. Fe^{3+} distribution in Hela cells measured by Prussian blue Staining

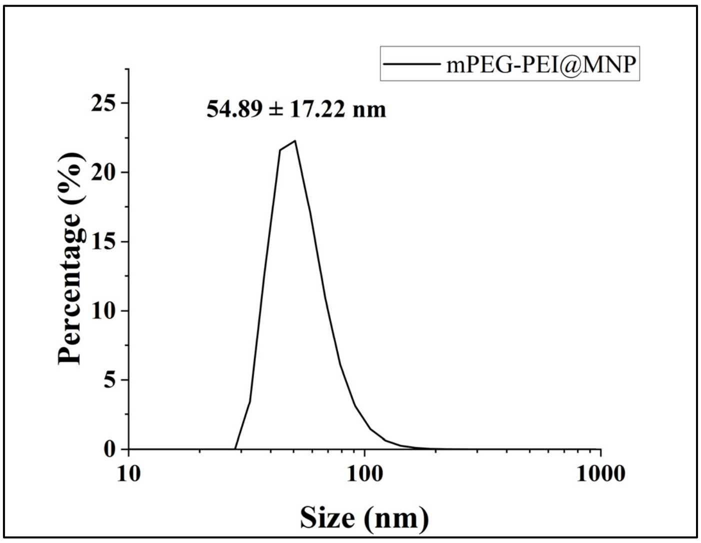

Figure S5. DLS distribution of mPEG-PEI@MNPs

Supplementary Methods

Method S1. Preparation of mPEG-PEI@MNPs (mPP@MNPs):

(1) The suspension of DMSA@MNPs (containing 5 mg of Fe), and 20 mg of methoxy-polyethylene glycol-polyethyleneimine (mPEG-PEI, Mw 2000–25000, Provided by Xi'an Ruixi Co. Ltd.) were mixed in 40 ml of pure water.

(2) The above mixture was reacted under electric stirring at 500 rpm with 100 W ultrasonic vibration for 1 h.

(3) Removal of excess mPEG-PEI: after step (2), the products were then purified by ultrafiltration (100 kDa NMWL) for 6 times, and kept in aqueous suspension. The DLS distribution of mPP@MNPs is shown in Figure S5.

Method S2. Measurement of Fe concentration by UV-Vis Spectrophotometry:

(1) 50 μL of the sample was added into 2 mL of hydrochloric acid (6 mol/L), and 100 W ultrasonic vibration for 30 min was used.

(2) 1 mL of hydroxylamine hydrochloride solution ($HONH_3Cl$, mass fraction 10%) was added, and then 100W ultrasonic vibration for 10 min was used.

(3) 2 mL of 1,10-phenanthroline solution (mass fraction 0.1%) was added and mixed well.

(4) 2 mL of NaOH solution (6 mol/L) was added and the solution was shacked quickly.

(5) 5 mL of pH 5.0 HAc-NaAc buffer (17 g of NaAc with 15 mL of glacial acetic acid, made up to 62.5 mL) was added. The solution was shacked well and placed for 30 min.

(6) The solution to be measured was made up to 50 mL with ultrapure water.

(7) Baseline and Background: Ultrapure water was taken into a quartz cuvette as the background for baseline scanning (520 nm–500 nm), and the absorbance measured at the wavelength of 510 nm was set as 0 value.

(8) Absorbance: The solution to be measured was added into a quartz cuvette, and the absorbance was measured at the wavelength of 510 nm.

(9) The Fe concentration was determined according to the standard curve.

Method S3. Prussian blue staining:

(1) Cells were fixed with 4% paraformaldehyde solution for 30 min at 4 °C.

(2) Staining solution preparation: 1M potassium ferrocyanide solution was mixed with 1 M hydrochloric acid in a 1:1 ratio.

(3) Paraformaldehyde solution was removed, and the cells were rinsed for 3 times with PBS buffer.

(4) Staining solution was added to the cells, and the cells were placed at room temperature for 30 min–60 min.

(5) Prussian Blue Staining Solution was removed, and the cells were rinsed for 3 times with PBS buffer.

(6) Nuclear Fast Red Staining Solution was used at room temperature for 3 min.

(7) Nuclear Fast Red Staining Solution was removed, and the cells were rinsed for 3 times with PBS buffer.

(8) Cells were observed under a light microscope.
